# Polyhedral 3D structure of human plasma very low density lipoproteins by individual particle cryo-electron tomography^1^[S]

**DOI:** 10.1194/jlr.M070375

**Published:** 2016-10

**Authors:** Yadong Yu, Yu-Lin Kuang, Dongsheng Lei, Xiaobo Zhai, Meng Zhang, Ronald M. Krauss, Gang Ren

**Affiliations:** Molecular Foundry,* Lawrence Berkeley National Laboratory, Berkeley, CA 94720; Atherosclerosis Research,† Children’s Hospital Oakland Research Institute, Oakland, CA 94609

**Keywords:** apolipoprotein B, antibodies, electron microscopy, three-dimensional

## Abstract

Human VLDLs assembled in the liver and secreted into the circulation supply energy to peripheral tissues. VLDL lipolysis yields atherogenic LDLs and VLDL remnants that strongly correlate with CVD. Although the composition of VLDL particles has been well-characterized, their 3D structure is elusive because of their variations in size, heterogeneity in composition, structural flexibility, and mobility in solution. Here, we employed cryo-electron microscopy and individual-particle electron tomography to study the 3D structure of individual VLDL particles (without averaging) at both below and above their lipid phase transition temperatures. The 3D reconstructions of VLDL and VLDL bound to antibodies revealed an unexpected polyhedral shape, in contrast to the generally accepted model of a spherical emulsion-like particle. The smaller curvature of surface lipids compared with HDL may also reduce surface hydrophobicity, resulting in lower binding affinity to the hydrophobic distal end of the N-terminal β-barrel domain of cholesteryl ester transfer protein (CETP) compared with HDL. The directional binding of CETP to HDL and VLDL may explain the function of CETP in transferring TGs and cholesteryl esters between these particles. This first visualization of the 3D structure of VLDL could improve our understanding of the role of VLDL in atherogenesis.

Lipoprotein particles are composed of amphipathic apolipoproteins, phospholipids, and cholesterol at their surfaces and neutral lipids, including TG and cholesteryl ester (CE), in their cores (1–3). Lipoproteins serve to transport lipids between tissues in the aqueous environment of the blood. Through ultracentrifugation, human plasma lipoproteins can be separated into HDLs, LDLs, VLDLs, and chylomicrons (4–8) in descending order of hydrated density. Different lipoproteins utilize different apolipoproteins as scaffolds upon which lipids associate. Specifically, HDL contains apoAI; LDL and VLDL contain apoB100; and chylomicrons contain apoB48, which corresponds to the N-terminal 48% of apoB100. apoB100, a 4,536 amino acid glycoprotein, is one of the largest single polypeptide chain proteins. VLDLs are assembled in the endoplasmic reticulum of liver parenchymal cells, where apoB100 is lipidated cotranslationally by TG and other lipids (9–14). After further intracellular lipidation and processing in the endoplasmic reticulum and Golgi, VLDLs are secreted into the circulation, where additional apolipoproteins, including apoAs (apoAI, apoAII, and apoAIV), apoCs (apoCI, apoCII, and apoCIII), and apoE, are acquired (8).

A major function of VLDLs is to transport TGs from the liver to peripheral tissues for use as an energy source (8, 15). The transfer process involves anchoring to endothelial surfaces by glycosylphosphatidylinositol-anchored HDL binding protein 1 (16) and activation of LPL by apoCII, resulting in the hydrolysis of VLDL TG, the release of free fatty acids, and the formation of VLDL remnant particles (17, 18). The remnants can be further hydrolyzed to form LDLs by hepatic lipase, and the LDLs can be internalized by several mechanisms, including interaction with the LDL receptor (8). In plasma, VLDLs can also exchange their containing TGs with HDL CEs mediated by CE transfer protein (CETP) via a tunnel mechanism (19–21) by which the hydrophobic distal end of the N-terminal β-barrel domain dominantly interacts with HDLs via a hydrophobic interaction (22). It is unclear why this hydrophobic distal end has less interaction with the same types of surface lipids of VLDL. The directional interaction of CETP with HDL and VLDL may relate to the directional transfer of TGs and CEs between VLDL and HDL. Cholesterol-enriched VLDL lipolytic remnants are associated with increased risk of CVD (23).

In plasma, VLDLs have highly complex compositions and the widest variation in particle size among the lipoprotein classes, with diameters ranging from 30 to 100 nm (7, 24, 25). Their heterogeneity poses a great challenge in studying their 3D structure via current structural biology methods, such as X-ray crystallography, nuclear magnetic resonance, or cryo-electron microscopy (cryo-EM) single particle reconstruction, which require either a 3D lattice or mono-dispersed particles of repeating structure. Although recent developments have enabled single particle reconstruction to classify the 3D structures of a few different conformations in silico, the numerous combinations of proteins and lipids among VLDL particles preclude a simple solution.

To understand the general 3D structure of VLDL particles and the variations among them, we imaged human plasma VLDL particles under near native conditions by cryo-electron tomography (cryo-ET) and then reconstructed the 3D density maps of each VLDL particle by the individual-particle electron tomography (IPET) reconstruction method (26). To confirm the 3D structures, we also examined the VLDLs below and above their lipid phase transition temperature and reconstructed the 3D density maps of the complexes of VLDLs bound to an anti-apoB100 antibody. Comparison of the structures indicated that VLDL surface proteins and phospholipids cooperate with each other to form a polyhedral structure.

## MATERIALS AND METHODS

### VLDL and antibody

Human VLDLs were isolated from the plasma of a healthy individual by standard overnight ultracentrifugation at d = 1.006 g/ml, 40,000 rpm, and 10°C. Mouse anti-human apoB monoclonal antibody, mAB012, was obtained from Chemicon (EMD Millipore Corporation, Temecula, CA). The epitope of mAB012 is the first 20 N-terminal amino acid residues of human apoB100. To make the antigen-antibody complex, VLDLs and mAB012 were mixed at a 1:1 molar ratio and incubated overnight at 4°C before plunge-freezing the cryo-EM grids.

### Cryo-EM sample preparation and data acquisition

Plunge-freezing of the cryo-EM grids was conducted using a Leica EM GP (Leica, Buffalo Grove, IL) that incorporates a chamber to control the humidity and temperature for blotting and evaporation. VLDL was first preincubated in a water-bath at 4, 40, and 45°C, the first temperature below and the latter two temperatures above the phase transition temperature (20–40°C), for more than 30 min. For each preincubation, the chamber of the plunge-freezer was set to the preincubation temperature, and the relative humidity in the chamber was maintained at 85%. Preincubated VLDL solution (3 μl) was applied to a glow-discharged lacey-carbon grid on a 200-mesh grid (EMS, Hatfield, PA) and kept in the chamber for 5 min prior to blotting and plunge-freezing.

Cryo-EM imaging was conducted using a Zeiss Libra 120 transmission electron microscope (Carl Zeiss SMT GmbH, Oberkochen, Germany) equipped with a LaB_6_ gun (operating at 120 kV), an in-column Ω energy filter, and a 4 k × 4 k Gatan UltraScan 4000 CCD camera. The opening angle of the electron source was set to 100 μrad to select only the central portion. The electron source was further constrained by a 75 μm-diameter condenser aperture, which shines on an area twice the size of the CCD at 50 k× (2.4 Å/pixel). A 50 μm-diameter objective aperture after the specimen was used to increase contrast. The in-elastically diffracted electrons were removed by using an in-column Ω energy filter (window width set to 20 eV).

A single-axis tilt series of frozen hydrated VLDLs alone was collected from −63° to +63° in steps of 1.5° at a nominal magnification of 50 k× (2.4 Å/pixel). The defocuses of the tilted views were set to 2 μm. The data were collected in low-dose mode, and the total dose was ∼150 e^−^/Å^2^. A single-axis tilt series of the VLDL-mAB012 complex was collected under a scheme similar to that of the VLDLs alone. To preserve the delicate structure of the antibodies, the total dose was reduced to ∼80 e^−^/Å^2^, and the micrographs were 2× binned during image acquisition (4.8 Å/pixel). Low-dose data acquisition was conducted by using the transmission electron microscope (TEM) tomography software (Gatan Inc., Pleasanton, CA) in advanced tomography mode.

### Data processing and 3D reconstruction

The tilt series of the micrographs was initially aligned using the software package, IMOD (27). The defocus values of the tilt series were calculated by using the programs tomops.exe and tomoctffind.exe in TomoCTF (28). The tilt views were phase-flipped by using the ctfcorrect.exe program of TomoCTF. To determine the 3D structures of individual particles, we boxed the images of the particle from each tilt series of micrographs and submitted the tilt series of the particle for reference-free alignment and 3D reconstruction by IPET (26). To validate the 3D reconstructions from IPET, a popular 3D reconstruction method, IMOD, was also used for reconstruction. In the absence of nano-gold particles, the centers of the small VLDL particles were used as fiducial markers to track and align the raw views of the tilt series. The fine alignment using these markers yielded an accuracy of 3 nm or better. A 3D whole tomogram was constructed from the aligned views using the weighted back-projection implemented in IMOD.

### Structural analysis

The resolution of the refined 3D model was estimated by the Fourier shell correlation (FSC) between two 3D density maps reconstructed independently from odd and even number tilt series, respectively. The resolution based on the 0.143 threshold or the 0.5 threshold is reported. The 3D structure was displayed by using UCSF Chimera (29). To gain the overall shape of each particle, the VLDL particles were simplified to polyhedrons by manually marking the vertices on the surface of 5.0 nm low-pass-filtered maps, connecting the vertices to represent the observed edges, and grouping the edges to represent the particle faces. These structural markers were chosen at a scale near 5.0 nm, leaving out smaller features for the sake of simplicity. Slight curvature of the faces was tolerated by only marking edges where dihedral angles were larger than 20° (the dihedral angle is defined as the angle between two nearby intersecting surface planes).

## RESULTS

### 2D images of VLDL particles by optimized negative stain and cryo-EM

A plasma VLDL sample prepared from a healthy person with a normal TG level of 127 mg/dl was examined by both optimized negative stain (OpNS) (30, 31) EM and cryo-EM techniques. OpNS was refined from conventional negative staining to prevent lipoprotein particles, especially HDLs, from stacking together (24, 30, 31). Cryo-EM is a cutting-edge technique used to examine proteins at near native state via imaging the proteins embedded in vitreous ice (32–34), which can avoid potential artifacts associated with negative staining, such as dehydration and flattening. However, the image contrasts are significantly lower than the contrasts of negative staining.

**Fig. 1.**
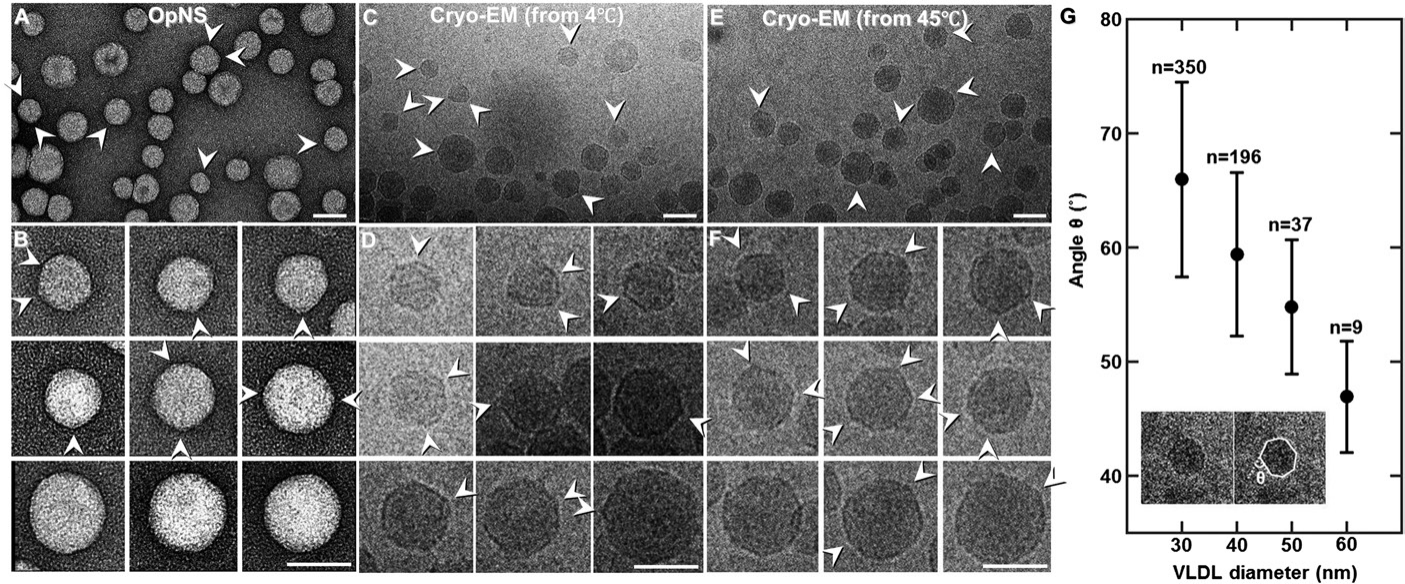
Electron micrographs of VLDL particles by negative-staining EM and cryo-EM. A: Survey view of the negative-staining VLDL particles. B: Representative negative-staining particles sorted by size. C: A field of view of VLDLs kept at 4°C before plunge-freezing. D: Representative particles at 4°C sorted by size. Background variation is because of differences in the thickness of the ice where the particles are embedded. E: Survey view of VLDLs kept at 40°C before plunge-freezing. F: Representative particles at 40°C sorted by size. Arrowheads indicate the vertices on the VLDL particles. G: In total, ∼600 VLDL particles are divided into 30, 40, 50, and 60 nm size groups (n is the number of particles in each group). The average smallest angles of these groups are 67, 60, 55, and 45°, respectively. The error bars represent the standard deviations. The inset indicates how the particle circumference is marked, and the smallest angle θ is taken. Scale bars: 50 nm (A, C, E); 20 nm (B, D, F).

Survey OpNS-EM micrographs (**Fig. 1A**) and selected particle views (Fig. 1B) displayed roundish shapes of the VLDL particles ranging from 30 to 60 nm in diameter. However, the smaller particles displayed more significant surface vertices (indicated by arrows in Fig. 1A, B), which differs from the generally accepted idea of spherical emulsion-like particles.

To confirm that the surface vertices were not induced by an artifact of the negative-staining method, the cryo-EM technique was used to examine the same sample frozen from a starting temperature of 4°C. The survey cryo-EM micrographs (Fig. 1C) and selected particle views (Fig. 1D) of VLDL embedded in vitreous ice showed that the particles have a similar diameter range to those observed with negative staining, i.e., from 30 to 60 nm. Also consistent was the finding that VLDL displayed angular shapes with several vertices on each particle, especially for small VLDL (indicated by arrows in Fig. 1C, D).

We next sought to investigate whether the angular shape is an intrinsic structural feature of VLDL or an artifact of the crystalline core of TGs induced by low temperature when the sample was frozen from 4°C, which is below the lipid phase transition temperature (∼20–40°C) (35). To do this, we repeated the above experiment after incubating the same sample at a temperature above the phase transition temperature (40–45°C for 30 min), directly flash freezing the sample from the above phase transition temperature into liquid nitrogen temperature by the cryo-EM technique, and examining the sample under the same cryo-EM operation conditions. The freezing speed is too fast (on the order of 10^4^–10^5^ K/s) for the water molecules to form a crystal (36, 37), and it is reasonable to assume that molecules larger than water, such as lipids, have insufficient time to crystallize during rapid freezing. Thus, structural changes, such as crystallization of TGs and CEs, during the freezing process are unlikely. The cryo-EM rapid freezing technique has been utilized since the early 1980s to preserve biological specimens in their native state for TEM examination, and to our knowledge, there are no reported freezing related artifacts (32–34). Thus, VLDL visualized by cryo-EM should be in its native structure and conformation.

The survey cryo-EM micrographs of VLDL particles frozen from above the phase transition temperature exhibited essentially the same particle diameter range and confirmed the angular morphologies as those frozen from below the phase transition temperature (Fig. 1E, F), i.e., the angular shape was more pronounced in smaller rather than larger VLDL particles. The consistency of the findings in the above experiments suggests that the angular shape is an inherent feature of the VLDL structure.

Statistical analysis of ∼600 cryo-EM images of VLDL particles showed that the particle diameter is linearly related to the surface angle. For particles within four size groups (30–39 nm, 40–49 nm, 50–59 nm, and 60–69 nm), the average smallest angles were 67, 60, 55, and 46°, respectively (Fig. 1G).

The high heterogeneity of plasma VLDLs was consistent with previous results from negative-staining (NS) EM (7, 24) and cryo-EM (25). The opaque appearance of VLDLs in cryo-EM might be attributable to high density matter on their surfaces, such as apolipoproteins.

### 3D reconstructions of six individual VLDL particles by IPET

**Fig. 2.**
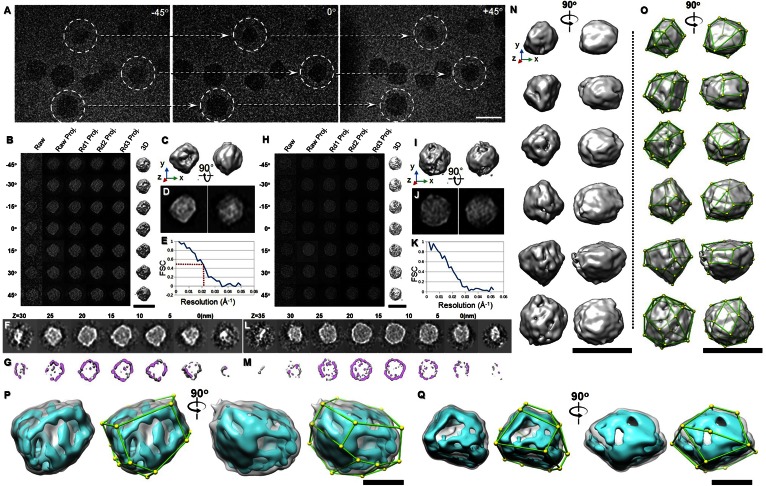
The 3D morphology of VLDL particles by cryo-ET. A: Three representative views of the single-axis tilt series of frozen hydrated VLDLs. B–G: Refinement procedures and results for one VLDL particle (image contrast reversed). B: IPET 3D reconstruction procedures. C, D: Two orthogonal views of refined particles low-pass filtered at 50 Å shown as an iso-surface representation (top) or a reprojection (bottom). The reader faces the Z-axis of the 3D reconstruction when viewing the left panel. E: Resolution was estimated by FSC between two models built from odd- and even-numbered views, respectively. F, G: The XY slices of the 3D maps at different heights are displayed as projection (top) and iso-surface (bottom) views. H–M: IPET 3D reconstruction procedures of another VLDL particle are shown. N: The 3D density maps of six representative VLDL particles are reconstructed and displayed from two perpendicular directions. The reader faces the Z-axis of the 3D reconstruction when viewing the left panel. Each map was low-pass filtered to 50 Å. O: The same maps as in (N) marked with the vertices and edges. P, Q: The 3D density maps of two representative VLDLs are displayed at a high contour level (cyan color) and a low contour level (gray color). The high contour map shows the structure of the high density components, e.g., apolipoproteins of VLDL. Scale bars: 50 nm (A–O); 25 nm (P, Q).

To confirm the observation of the angular shape of VLDL in 3D, we imaged the samples from a series of tilting angles by cryo-ET under low-dose mode with a total dose of ∼150 e^−^/Å^2^ and a magnification of 50 k× (corresponding to 2.4 Å/pixel) (supplemental Video S1). The survey of cryo-ET micrographs at three representative angles displayed eight VLDL particles whose diameters and shapes differed substantially from each other, such that they could not be averaged for 3D reconstruction (**Fig. 2A**); therefore, we used the IPET reconstruction method we developed to reconstruct the 3D density maps for single VLDL particles.

For IPET 3D reconstruction, a series of tilted images of each targeted particle was boxed from the tilted whole cryo-ET micrographs after contrast transfer function correction. The selected tilt images of a representative targeted particle were iteratively aligned to their global center to achieve a final ab initio 3D reconstruction (Fig. 2B, left panel). The step-by-step refinement procedures and the intermediate results are shown in Fig. 2B. The final 3D density map (after low-pass filtering to 5.0 nm) displayed from two perpendicular viewing directions showed the particle having a diameter of 35 nm with an overall polyhedral structure (Fig. 2C), which was confirmed by the corresponding 2D projections (Fig. 2D). The FSC analysis showed that the 3D resolution is ∼3.5 nm based on a 0.143 criteria or ∼5.0 nm based on a 0.5 criteria (Fig. 2E) (details given in the Materials and Methods). The projections of the slices of 3D density maps at different heights (Fig. 2F, G) showed that the density of the core was generally lower than that of the shell and that the shell was ∼3 nm thick on average, consistent with the thickness of a phospholipid monolayer. The shell was uneven, even when low-pass filtered at 5.0 nm, indicating an irregular distribution of protein densities in the lipid membrane.

The achieved resolution of 3.5–5 nm seems to be incompatible with the observation of a phospholipid monolayer (∼3 nm). Several reasons may be related to this phenomenon: *i*) The resolution of 3.5–5 nm is an estimated resolution based on the FSC analysis of two 3D reconstructions in which each of the 3D reconstructions was reconstructed from half of the tilt images. Thus, the qualities of these two 3D reconstructions could be poorer than the final 3D reconstruction that incorporates the full set of tilted images, especially when the total number of tilt images is less than 100, which leads to an underestimation of the final 3D resolution. *ii*) The resolution estimated from the FSC analysis is defined differently from that of the standard optical resolution, i.e., the distance between two distinguishable radiating points. The FSC resolution is a rough estimated resolution. *iii*) Identifying a single line object (lipid monolayer) is easier than identifying two distinct points. Identifying the location of the single line object is dependent only on the intensity of the object and not on the dimension of the object. Based on the above reasons, the lipid monolayer, which forms a line object, can be observed under the current resolution.

By repeating the above IPET process, we reconstructed a second 3D density map from another individual VLDL particle (Fig. 2H–M). The representative tilt images showed that the VLDL was still visible (Fig. 2H, left panel). Through IPET reconstruction processing, the tilt images were gradually and iteratively aligned to the global center (Fig. 2H). The two perpendicular views of the final 3D density maps of the second VLDL particle showed that it is a near roundish structure of 40 nm in diameter, but still has a visible polyhedral structure (Fig. 2I). The shape can also be displayed by its corresponding projections (Fig. 2J). The observed high density shell and low density core of the VLDL particle was confirmed by the projections of the slices of the 3D density map at different heights (Fig. 2L, M).

**Fig. 3.**
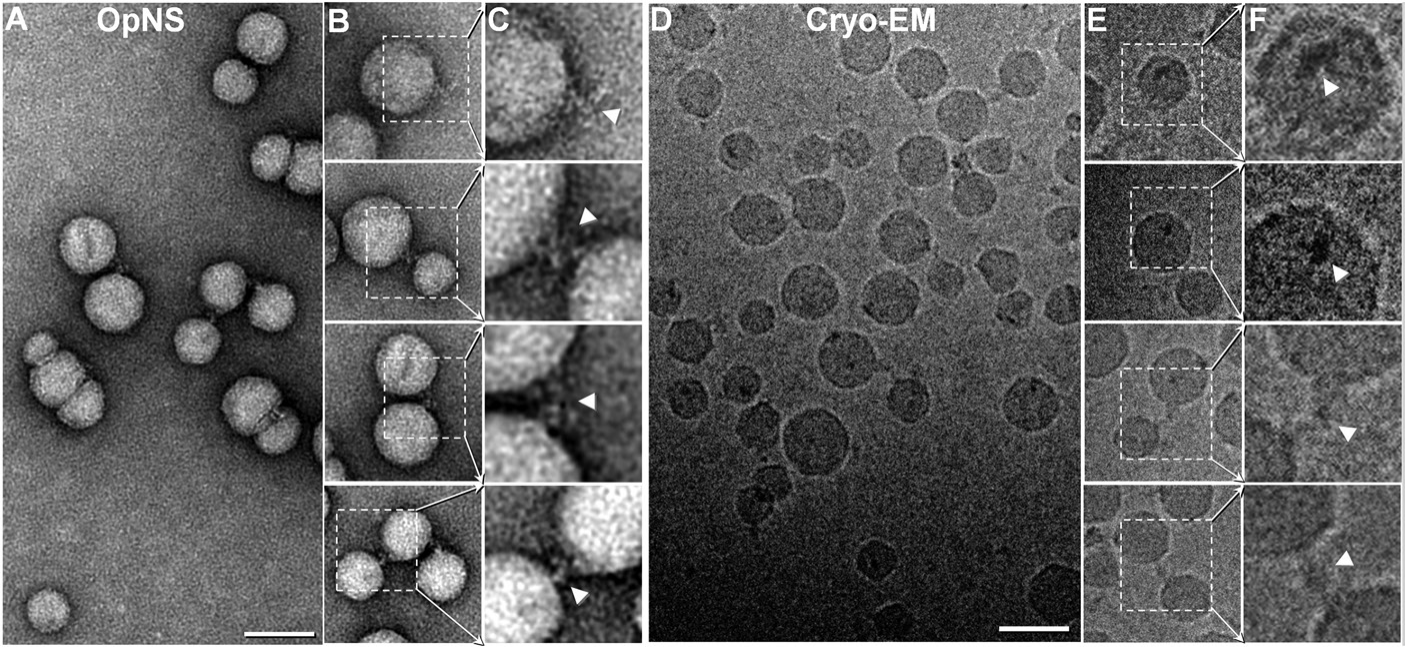
The 2D images of negative-staining and frozen VLDL complex with mAB012. A: Survey view of a negative-staining VLDL and mAB012 mixture. B: Representative VLDL particles bound with an antibody. C: Zoomed-in view of (B) showing the antibody details. D: A field of view of the same VLDL and mAB012 mixture except that this shows a frozen hydrated sample that used the cryo-EM plunge-freezing technique. E: Representative VLDL particles bound by an antibody. F: Zoomed-in view of (E) showing the antibody details. Scale bars, 50 nm.

Through particle-by-particle 3D reconstructions, a few tens of VLDL-antibody complex particles were reconstructed. Six representative particles in increasing sizes are displayed at two directions that differ by 45° around the vertical axis (Fig. 3N). The resolutions of the final 3D reconstructions were ∼5.0 nm, which allowed us to model the particles’ surface shapes (Fig. 3O). The refined 3D density maps indicated obviously flat faces and the neighboring flat faces interacted to each other forming dihedral angles. The shapes can be modeled as polyhedrons by manually marking the vertices, connecting the vertices to represent the observed edges, and grouping the edges to represent the particle faces (Fig. 2N, O). The densities at the edges were generally higher than the densities on the surfaces (Fig. 2P, Q). In this model, only the dihedral angles larger than 20° were marked as edges, and therefore, a slight curvature of the faces was tolerated. By this method, there were more than 10 flat faces on each particle, in which a few dihedral angles were at or near 90°.

### 3D reconstructions of six individual VLDL-antibody complexes by IPET

We next sought to confirm that the observed polyhedral 3D particles contained the major VLDL structural protein, apoB100. We therefore repeated the above experiments after incubating the sample with the monoclonal apoB antibody, mAB012, at 4°C overnight. The incubation mixture was then examined by both OpNS and cryo-EM (**Fig. 3**). The survey OpNS-EM micrograph and zoomed-in views of the particles showed primarily roundish particles attached to the Y-shaped density of the antibody (Fig. 3A–C). Notably, a significant number of roundish particles were linked to each other by the Y-shaped density of the antibody, suggesting that both Fab domains in an antibody could bind to apoB100 on the VLDL particles. A roundish particle attached with two Y-shaped antibodies was not observed, confirming that the binding of this antibody is specific to VLDL and that each VLDL particle contains one apoB100, as expected.

To confirm that the antibody labeling was not an artifact of the negative-staining method, the cryo-EM technique was used to examine the same sample. The survey cryo-EM micrographs and zoomed-in images confirmed surface binding of the roundish particles to densities with dimensions similar to those of the antibody. In several instances, the antibody-like densities each bridged a pair of roundish particles, forming a ternary complex, suggesting that these particles were bound to the antibody rather than incidentally overlapping with it. These results confirm that the roundish particles contain apoB100 and are very likely VLDLs. Notably, the smaller particles have a clearly angular shape, consistent with the morphology of VLDL unbound to apoB100 under cryo-EM conditions.

**Fig. 4.**
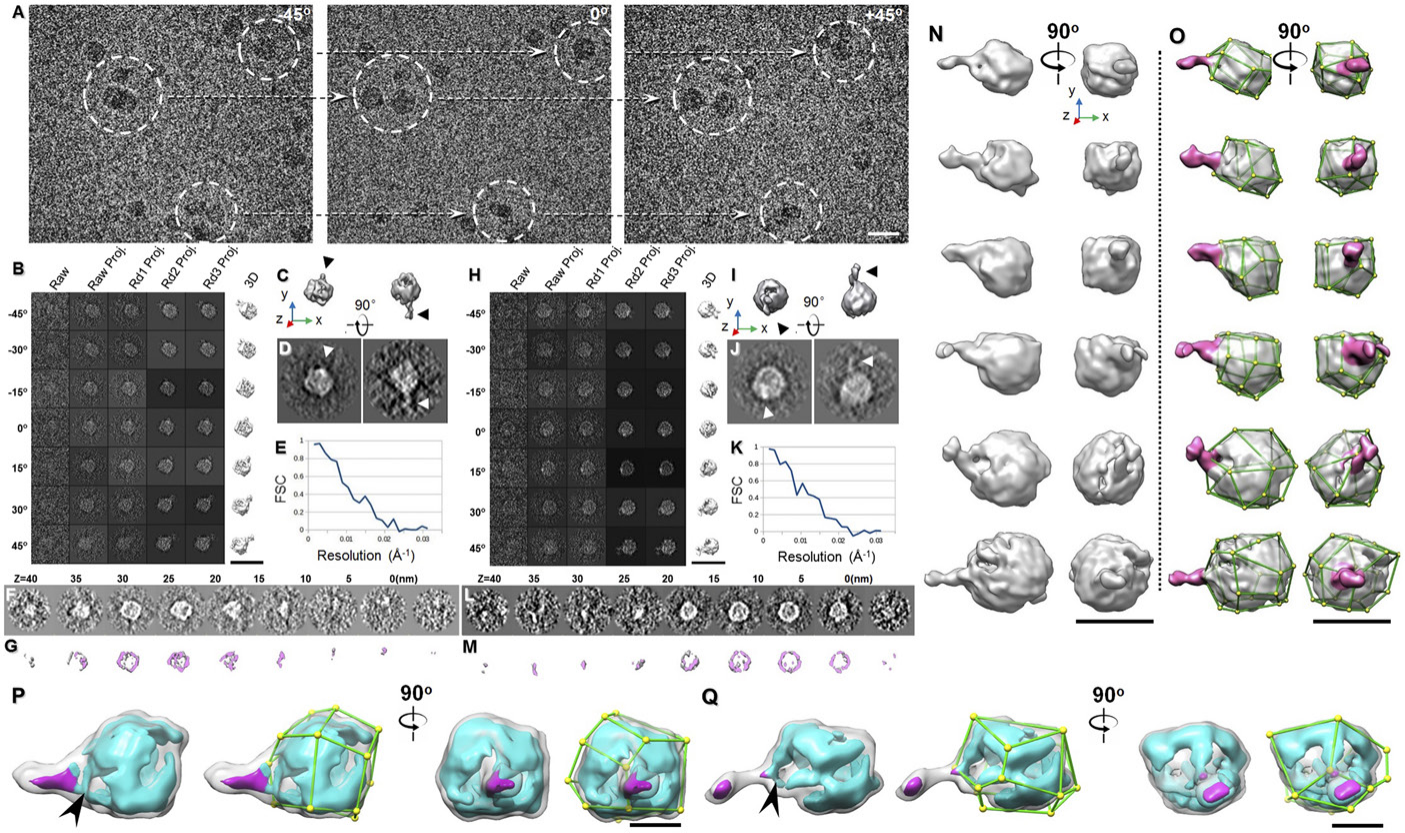
The 3D tomogram of the VLDL-mAB012 complex by cryo-ET. A: Three representative views of the single-axis tilt series of frozen hydrated VLDL and antibody mAB012 mixtures. B–G: Refinement procedures and results from one VLDL-antibody complex (image contrast reversed). B: IPET refinement procedures. C, D: Two orthogonal views of refined particles low-pass filtered at 50 Å shown as an iso-surface representation (top) and a reprojection (bottom). The reader faces the Z-axis of the 3D reconstruction when viewing the left panel. E: Resolution was estimated by FSC between two models built from odd- and even- numbered views, respectively. F, G: The XY slices of the particle at different heights are displayed as projection (top) and iso-surface representations (bottom). H–M: IPET 3D reconstruction procedures for another VLDL-antibody complex. N: The 3D density maps of six representative VLDL-antibody complexes displayed from two perpendicular directions (rotated around the Y-axis by 90°). The maps were low-pass filtered to 50 Å. O: The same maps as in (N) but marked with the vertices and edges. The antibody densities are colored in pink. P, Q: The 3D density maps of two representative VLDL-antibody complexes are displayed at a high contour level (cyan color) and a low contour level (gray color), while the antibody portion is colored in magenta. The high contour map shows the structure of the high density components, e.g., apolipoproteins of VLDL. The arrows indicate the antibody binding area in the VLDLs. Scale bars: 50 nm (A–O); 25 nm (P, Q).

To further confirm that antibody-bound VLDL particles have a polyhedral 3D structure, we imaged the sample from a series of tilting angles by cryo-ET under a low-dose condition, i.e., a total dose of ∼80 e^−^/Å^2^, representing a dose much lower than that used for VLDL alone to preserve the delicate antibody structure (supplemental Video S1). The survey cryo-ET micrographs of VLDL particles at three representative angles displayed that 12 VLDL particles each have a unique diameter and shape, which could not be averaged together for 3D reconstruction (**Fig. 4A**). We therefore used the IPET reconstruction method we developed to reconstruct the 3D density map from each individual VLDL-mAB012 complex.

In the IPET 3D reconstruction, a series of tilted images of a targeted complex were boxed from the tilted whole cryo-ET micrographs after contrast transfer function correction. The representative tilt images showed that the complex was difficult to visualize (Fig. 4B, left panel). Through IPET reconstruction processing, the tilt images were gradually and iteratively aligned to their global center to achieve a final ab initio 3D reconstruction (Fig. 4B). The final 3D density map (after low-pass filtering to 5.0 nm) displayed from two perpendicular viewing directions showed a polyhedral-shaped particle 30 nm in diameter with an ∼10 nm protrusion representing the bound antibody (Fig. 4C). The protrusion and polyhedral shape were confirmed by the corresponding 2D projections (Fig. 4D). The observation of the linear-shaped antibody may be due to the low 3D resolution or may be because the antibody was undergoing a large-scale conformational change after binding to the antigen, such as that observed for antibodies bound to peptides via their Fab domains (38). FSC analysis showed that the 3D resolution was ∼6.0 nm based on the 0.143 criteria of FSC or ∼8.0 nm based on the 0.5 criteria (Fig. 4E). The projections of the slices of the 3D density map at different heights also confirmed the observed high density shell and low density core, as observed for the VLDL particles. In addition, the density of the antibody is roughly visible (Fig. 4F, G).

Considering the ∼10 nm protrusions that correspond to the antibody, the 3–4 nm thickness of the protrusion seems to be below the capability of a 6–8 nm resolution. The reasons for this observation are essentially similar to those described above regarding the VLDL surface lipid monolayer, i.e., the linear object can be observed under the current resolution.

By repeating the above IPET process, we reconstructed a second 3D density map from another individual VLDL bound with an antibody (Fig. 4H–M). As in the previous case, the representative tilt images showed that the VLDL was difficult to visualize (Fig. 4H, left panel). Through the IPET reconstruction processing, the tilt images were gradually and iteratively aligned to their global center (Fig. 4H). The perpendicular views of the final 3D density maps of the second targeted complex showed a polyhedral particle of 40 nm in diameter with a protrusion ∼10 nm in length. The complex has a visible polyhedral structure (Fig. 4I). The shape and surface antibody protrusion can also be visualized by their corresponding projections (Fig. 4J) and by the projections of the central slices and their 3D slices of the density map (Fig. 4L, M). The high density shell and low density core of the VLDL particle can also be confirmed. Moreover, the density of the antibody is also roughly visible.

Through particle-by-particle 3D reconstructions, a few tens of VLDL-antibody complex particles were reconstructed. Six representative particles with increasing sizes are displayed from two perpendicular viewing directions (Fig. 4N). The resolutions of the final 3D reconstructions were ∼6.0 nm, which allowed us to model the particles’ surface shapes (Fig. 4O). The first four complexes have a diameter of ∼30 nm, and the last two complexes have diameters of 40 and 50 nm, respectively. The protrusion sizes were all ∼10 nm in length on these six density maps, and the antibody densities lengthwise are all perpendicular to VLDL. Particles in the 30 nm size group present more obvious polygonal shapes than those over 40 nm, as indicated by surface modeling (Fig. 4N, O), which is consistent with the morphology of VLDL alone.

Monoclonal antibodies against different parts of the apoB100 protein have been used to identify the location of apoB domains on LDL surfaces (39, 40). Similarly, the locations where the antibodies against apoB bind can indicate the position of apoB100 domains on VLDL. We have shown that the edges of VLDL particles are lined with high densities, which is presumably mainly due to apoB100 (Fig. 2P, Q). The mAB012 antibody bound in the 3D maps of the VLDL-antibody complexes showed that most antibody densities attach to the edges or vertices of polyhedral VLDL. None was found on VLDL faces. Figure 4P, Q shows the 3D maps of two representative VLDL-antibody complexes displayed at two contour levels. The high contour maps clearly indicate that antibodies bind directly to the edges (arrows in Fig. 4P, Q).

The benefit of using a divalent antibody instead of a monovalent antibody (e.g., Fab fragments) to label VLDL is that a divalent antibody can bind two VLDL particles simultaneously, which can confirm whether the antibody can bind to VLDL via antibody-antigen interactions rather than through nonspecific binding. The frequent observation of a divalent antibody bridging two VLDLs (Fig. 3A–D) indirectly indicates that the antibody used was functional. Moreover, whether cryo-EM 3D reconstruction of a single antibody can be reconstructed is largely unclear (more details in the Discussion section), and the use of even smaller antibodies, e.g., a monovalent antibody, would be more challenging or more speculative.

Taken together, the studies of VLDL alone and of the VLDL-antibody complex suggest that a polyhedral 3D structure is an intrinsic property of VLDL particles, that this feature is independent of the phase transition of core lipids, and that it is not affected by apoB100 antibody binding.

## DISCUSSION

In this study, we used multiple TEM techniques, including OpNS, cryo-EM, and cryo-ET, to examine human plasma VLDL particles above and below their neutral lipid phase transition temperature. The EM images showed that the VLDL particles exhibited considerable heterogeneity in size and shape. Importantly, cryo-ET 3D reconstructions of individual VLDL particles and VLDL-antibody complexes revealed a polyhedral structure, which was most evident among smaller-sized VLDLs. The tendency to be polyhedral and to show obvious flat faces on smaller VLDL particles seems to converge to the discoidal shape of LDL with two large opposing flat faces.

### Validation of 3D reconstructions of cryo-ET

Cryo-ET has recently demonstrated its ability to be used to develop 3D reconstructions at a resolution better than 2 nm by averaging hundreds to thousands of sub-volumes, such as for reconstruction of the 26S proteasome (41) and the nuclear pore complex (42). Although this method can increase the 3D reconstruction resolution by averaging out noise, it also introduces the possibility of averaging both the errors of the reconstruction and the flexible portions of the molecules (which cannot be distinguished from the noise or error of the reconstruction in each of the sub-volumes). Thus, 3D reconstruction from an individual protein particle would be a fundamental advancement in the study of flexible protein structures and fluctuations. Recently, we reported the IPET reconstruction method (26) and used this method to reconstruct 120 density maps at 1–2 nm resolution from 120 individual IgG1 antibody molecules (43). This method was also used to reconstruct density maps from 14 individual particles of 84 base-paired double-strand DNA conjugated with 5 nm Nanogold (44) from negative-stained samples. Whether the 3D density map of a small and flexible protein, such as an antibody, can be reconstructed by cryo-ET remains an open question. Although, the first cryo-ET 3D reconstruction of an individual IgG antibody was reported a decade ago using the COMET reconstruction method (45, 46), the previous studies did not show raw data or intermediate results nor did they include all of the final 3D reconstructions for the readers to evaluate the results. Moreover, a publication of the antibody binding protein using the same COMET approach was retracted because more than 90% of the reconstructions were invalid based on a validation using the third-party reconstruction IMOD method (47, 48)

**Fig. 5.**
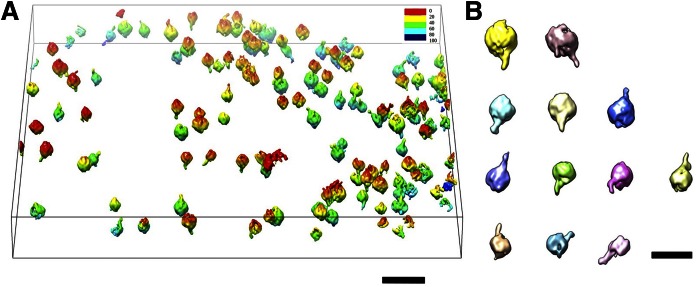
Validation of the 3D reconstructions of cryo-ET VLDL-antibody complexes by a third party software, IMOD. The whole tomogram, which was low-pass filtered at 50 Å, is represented as an iso-surface view and is ramp-colored from red to blue by height. The whole tomogram contains approximately 100 VLDL particles with ∼80% bound to antibodies (mAB012). The high binding efficiency of the antibodies to VLDL in this equimolar ratio mixture indicates the high antibody specificity and confirms that these particles contain apoB protein. The VLDL-antibody complexes are each shaped like a garlic bulb, in which the antibody resembles an ∼10 nm-long stem, whereas the VLDL is shaped like a sphere with a 30 nm or larger diameter. Several ternary complexes exist, for which one antibody bridges two VLDL particles in each. Scale bars: 100 nm (A); 50 nm (B).

To validate our 3D reconstructions by a third-party method, we processed the same data for 3D reconstruction by IMOD (**Fig. 5**). The 3D reconstruction generated using IMOD (after low-pass filtering at 5.0 nm) showed that the VLDL-mAB012 complexes have a garlic bulb shape, in which the antibody resembled a ∼10 nm-long stem, whereas the VLDL was observed approximately as a sphere of 30 to ∼100 nm in diameter (Fig. 5). Among a total of ∼100 VLDL particles, ∼80% were bound with antibodies. The high binding efficiency of the antibody to VLDL in this equimolar ratio mixture indicates the high specificity of this antibody and confirms that these particles primarily contain apoB protein (Fig. 5). The results are consistent with our 3D reconstructions, suggesting that a bound antibody on a VLDL particle can be reconstructed by cryo-ET.

One may notice that the 3D reconstructions by IMOD (Fig. 5) display more spheroids that do not include an obvious polyhedral shape corresponding to the VLDL portion, which seems inconsistent with the angular shapes of VLDL shown in the IPET 3D reconstructions. Considering the angular shapes presented in the raw survey micrographs (Fig. 1C, E; Fig. 3D), the tilt images (Figs. 2A, 4A), and the movies of the tilt-series image (supplemental Video S1), the presence of the angular shape in the IPET 3D reconstructions instead of in the IMOD 3D reconstructions indirectly suggests that the IPET method may have a higher resolution capability. This benefit may be a result of our unique strategy employed in the IPET 3D reconstruction method, in which small-sized images instead of large micrographs were used for the 3D reconstruction to reduce the influence of image distortion (26).

Additionally, the 3D reconstructions from both IMOD and IPET had a less significant effect arising from the missing-wedge, causing a limited range of tilt angles of the specimen holder and missing data at these viewing angles. We discussed previously that the effect of the missing wedge is limited with small and thin objects, such as macromolecules instead of cell sections or a whole bacteria, when the tilted images were precisely aligned (26). The limited influence of the missing wedge has also been observed in IPET 3D reconstructions of other macromolecules, such as 160 kDa antibodies (38, 43), a 53 kDa cholesterol ester transfer protein bound to a liposome (22), and an 84 base-pair dsDNA bound to two 5 nm Nanogold particles (44).

### Surface lipid structure

The 3D reconstruction of VLDLs showed that the flat surface has a relatively lower density than the edges, suggesting that the surface low density is mainly comprised of lipids, while the edges are mainly formed by amphipathic apolipoproteins. The polyhedral structure of VLDL is not dependent on the phase transition of the core neutral lipids, as demonstrated by the examination of VLDL frozen at different temperatures. When the surface lipids form a flat structure, the chance of their fatty acid chains being exposed to the solution is greatly reduced, especially for small VLDLs.

A similar phenomenon of angular-shaped lipid surfaces was observed with 1,2-dimyristoyl-*sn*-glycero-3-phosphocholine (DMPC)-based liposomes frozen from below their phase transition temperature (4°C) and visualized by cryo-EM (49). The shorter fatty acid chains of DMPC compared with those of VLDL surface lipids (mainly POPC) are likely to form a plane by themselves; however, whether the angular structure can be retained above the phase transition temperature is unknown. A similar angular structure of VLDLs was also apparent in data from an earlier cryo-EM study; however, the angular structure was not specifically noted (50).

The hydrophobic edges of the planar surface lipids in the polyhedral VLDLs could be protected by VLDL containing apolipoproteins, although it is unclear how the hydrophobic edges of the planar surface DMPC lipids in the liposome vesicles without apolipoproteins are maintained in solution. There may be many reasons for this phenomenon. First, DMPC vesicles have a lipid bilayer, while VLDLs have a lipid monolayer. The inner layer lipids may move and change their orientation to protect the hydrophobic surface. Second, the polyhedral DMPC vesicle is shorter in fatty acid chains than in the VLDL major lipids, such as POPC. Shorter fatty acid chains may generate a smaller gap at the edges under the same dihedral angle between two planar lipid surfaces. Smaller gaps lead to less hydrophobicity and allow the polyhedral shape of the DMPC vesicle to be more easily maintained in solution.

### Potential biologic relevance of the polyhedral structure of VLDLs

Our study demonstrated that VLDL particles, especially smaller particles (∼30 nm diameter), exhibit flat faces and sharp edges, for which the dihedral angles could be as sharp as 90°. The flat faces of VLDL implicate low surface curvature of the outer shell phospholipid monolayer, which is different from the generally believed spherical shape due to the lack of a method to obtain the 3D structure from each heterogeneous VLDL particle. Our hypothesis is that the flat surface lipids of VLDL provide a relatively lower hydrophobicity than curved lipid surfaces, such as that of HDL. As a result, the N-terminal distal domain of CETP has less binding affinity to the flat surface lipid plane of polyhedral VLDLs than to the curved surface of spherical HDL. This hypothesis is consistent with the hydrophobic distal end of the N-terminal β-barrel domain of CETP interacting with HDL surface lipids and initializing CE uptake from the HDL core, as suggested by molecular dynamics simulations (21, 44, 51). It is also consistent with the observation that most CETPs in plasma bind to HDLs instead of VLDLs, which is due to the lower surface curvature (less hydrophobicity) of VLDLs, although the surface lipids of VLDLs are the same as those of HDLs. This directional binding of CETP may facilitate CETP-mediated CE transfer from HDL to VLDL.

Because the N-terminal β-barrel domain of CETP facilities the hydrophobic interactions with surface lipids for initializing CE uptake (21, 44, 51), we predicted the LDL surface could have more binding affinity to the N-terminal β-barrel domain of CETP that is between its binding affinities for HDL and VLDL because of the partially flat surface and partially curved surface revealed by the cryo-EM studies (25, 52–55). The intermediate level of binding affinities between HDL and VLDL may explain how CETP can transfer CE from HDL to LDL and from LDL to VLDL.

In summary, our cryo-ET 3D reconstruction without averaging different molecules demonstrates that VLDL particles have polyhedral 3D structures with large low density cores encapsulated by high density shells that are a few nanometers thick. The VLDL surface is composed of several flat faces conjugated by high density edges, suggesting that the VLDL surface is covered with flat lipids that adhere to amphiphilic apolipoproteins.

## Supplementary Material

Supplemental Data

## References

[1] van der Vusse G. J., Glatz J. F., Stam H. C., and Reneman R. S. 1992 Fatty acid homeostasis in the normoxic and ischemic heart. Physiol. Rev. 72: 881–940. 1438581 10.1152/physrev.1992.72.4.881

[2] Davis R. A., Engelhorn S. C., Pangburn S. H., Weinstein D. B., and Steinberg D. 1979 Very low-density lipoprotein synthesis and secretion by cultured rat hepatocytes. J. Biol. Chem. 254: 2010–2016. 217875

[3] McEneny J., O’Kane M. J., Moles K. W., McMaster C., McMaster D., Mercer C., Trimble E. R., and Young I. S. 2000 Very low density lipoprotein subfractions in Type II diabetes mellitus: alterations in composition and susceptibility to oxidation. Diabetologia. 43: 485–493. 10819243 10.1007/s001250051333

[4] Baynes J., and Dominiczak M. H. 2009. Medical Biochemistry. Elsevier, London.

[5] Durrington P. 2003 Dyslipidemia. Lancet. 362: 717–731. 12957096 10.1016/S0140-6736(03)14234-1

[6] Rifai N., Warnick G. R., and Dominiczak M. H. 2000 Handbook of Lipoprotein Testing. 2nd edition. AACC Press, Washington, DC.

[7] Vance D. E., and Vance J. E. 2002 Assembly and secretion of lipoproteins. *In* Biochemistry of Lipids, Lipoproteins, and Membranes. Elsevier, Amsterdam. 505–526.

[8] Dominiczak M. H., and Caslake M. J. 2011 Apolipoproteins: metabolic role and clinical biochemistry applications. Ann. Clin. Biochem. 48: 498–515. 22028427 10.1258/acb.2011.011111

[9] Alexander C. A., Hamilton R. L., and Havel R. J. 1976 Subcellular localization of B apoprotein of plasma lipoproteins in rat liver. J. Cell Biol. 69: 241–263. 177430 10.1083/jcb.69.2.241 PMC2109679

[10] Jamil H., Gordon D. A., Eustice D. C., Brooks C. M., Dickson J. K. Jr., Chen Y., Ricci B., Chu C. H., Harrity T. W., Ciosek C. P. Jr., 1996 An inhibitor of the microsomal triglyceride transfer protein inhibits apoB secretion from HepG2 cells. Proc. Natl. Acad. Sci. USA. 93: 11991–11995. 8876250 10.1073/pnas.93.21.11991 PMC38171

[11] Olofsson S. O., Asp L., and Boren J. 1999 The assembly and secretion of apolipoprotein B-containing lipoproteins. Curr. Opin. Lipidol. 10: 341–346. 10482137 10.1097/00041433-199908000-00008

[12] Shelness G. S., Ingram M. F., Huang X. F., and DeLozier J. A. 1999 Apolipoprotein B in the rough endoplasmic reticulum: translation, translocation and the initiation of lipoprotein assembly. J. Nutr. 129: 456S–462S. 10064309 10.1093/jn/129.2.456S

[13] Bakillah A., Nayak N., Saxena U., Medford R. M., and Hussain M. M. 2000 Decreased secretion of ApoB follows inhibition of ApoB-MTP binding by a novel antagonist. Biochemistry. 39: 4892–4899. 10769147 10.1021/bi9924009

[14] Ohsaki Y., Cheng J., Suzuki M., Fujita A., and Fujimoto T. 2008 Lipid droplets are arrested in the ER membrane by tight binding of lipidated apolipoprotein B-100. J. Cell Sci. 121: 2415–2422. 18577578 10.1242/jcs.025452

[15] Niu Y. G., and Evans R. D. 2011 Very-low-density lipoprotein: complex particles in cardiac energy metabolism. J. Lipids. 2011: 189876. 21773049 10.1155/2011/189876 PMC3136095

[16] Fang L., Choi S. H., Baek J. S., Liu C., Almazan F., Ulrich F., Wiesner P., Taleb A., Deer E., Pattison J., 2013 Control of angiogenesis by AIBP-mediated cholesterol efflux. Nature. 498: 118–122. 23719382 10.1038/nature12166 PMC3760669

[17] Eisenberg S., and Sehayek E. 1995 Remnant particles and their metabolism. Baillieres Clin. Endocrinol. Metab. 9: 739–753. 8593123 10.1016/s0950-351x(95)80113-8

[18] Cohn J. S., Marcoux C., and Davignon J. 1999 Detection, quantification, and characterization of potentially atherogenic triglyceride-rich remnant lipoproteins. Arterioscler. Thromb. Vasc. Biol. 19: 2474–2486. 10521378 10.1161/01.atv.19.10.2474

[19] Tall A. R. 1986 Plasma lipid transfer proteins. J. Lipid Res. 27: 361–367. 3522782

[20] Ihm J., Quinn D. M., Busch S. J., Chataing B., and Harmony J. A. 1982 Kinetics of plasma protein-catalyzed exchange of phosphatidylcholine and cholesteryl ester between plasma lipoproteins. J. Lipid Res. 23: 1328–1341. 7161562

[21] Zhang L., Yan F., Zhang S., Lei D., Charles M. A., Cavigiolio G., Oda M., Krauss R. M., Weisgraber K. H., Rye K. A., 2012 Structural basis of transfer between lipoproteins by cholesteryl ester transfer protein. Nat. Chem. Biol. 8: 342–349. 22344176 10.1038/nchembio.796 PMC3792710

[22] Zhang M., Charles R., Tong H., Zhang L., Patel M., Wang F., Rames M. J., Ren A., Rye K. A., Qiu X., 2015 HDL surface lipids mediate CETP binding as revealed by electron microscopy and molecular dynamics simulation. Sci. Rep. 5: 8741. 25737239 10.1038/srep08741 PMC4348656

[23] Mahley R. W., and Huang Y. 2007 Atherogenic remnant lipoproteins: role for proteoglycans in trapping, transferring, and internalizing. J. Clin. Invest. 117: 94–98. 17200713 10.1172/JCI30889 PMC1716223

[24] Zhang L., Song J., Cavigiolio G., Ishida B. Y., Zhang S., Kane J. P., Weisgraber K. H., Oda M. N., Rye K. A., Pownall H. J., 2011 Morphology and structure of lipoproteins revealed by an optimized negative-staining protocol of electron microscopy. J. Lipid Res. 52: 175–184. 20978167 10.1194/jlr.D010959 PMC2999936

[25] van Antwerpen R., La Belle M., Navratilova E., and Krauss R. M. 1999 Structural heterogeneity of apoB-containing serum lipoproteins visualized using cryo-electron microscopy. J. Lipid Res. 40: 1827–1836. 10508202

[26] Zhang L., and Ren G. 2012 IPET and FETR: experimental approach for studying molecular structure dynamics by cryo-electron tomography of a single-molecule structure. PLoS One. 7: e30249. 22291925 10.1371/journal.pone.0030249 PMC3265479

[27] Kremer J. R., Mastronarde D. N., and McIntosh J. R. 1996 Computer visualization of three-dimensional image data using IMOD. J. Struct. Biol. 116: 71–76. 8742726 10.1006/jsbi.1996.0013

[28] Fernández J. J., Li S., and Crowther R. A. 2006 CTF determination and correction in electron cryotomography. Ultramicroscopy. 106: 587–596. 16616422 10.1016/j.ultramic.2006.02.004

[29] Pettersen E. F., Goddard T. D., Huang C. C., Couch G. S., Greenblatt D. M., Meng E. C., and Ferrin T. E. 2004 UCSF Chimera–a visualization system for exploratory research and analysis. J. Comput. Chem. 25: 1605–1612. 15264254 10.1002/jcc.20084

[30] Rames M., Yu Y., and Ren G. 2014 Optimized negative staining: a high-throughput protocol for examining small and asymmetric protein structure by electron microscopy. J. Vis. Exp. 90: e51087. 25145703 10.3791/51087 PMC4710468

[31] Zhang L., Song J., Newhouse Y., Zhang S., Weisgraber K. H., and Ren G. 2010 An optimized negative-staining protocol of electron microscopy for apoE4 POPC lipoprotein. J. Lipid Res. 51: 1228–1236. 19965615 10.1194/jlr.D002493 PMC2853450

[32] Gilkey J. C., and Staehelin L. A. 1986 Advances in ultrarapid freezing for the preservation of cellular ultrastructure. J. Electron Microsc. Tech. 3: 177–210.

[33] Bailey S. M., Chiruvolu S., Longo M. L., and Zasadzinski J. A. 1991 Design and operation of a simple environmental chamber for rapid freezing fixation. J. Electron Microsc. Tech. 19: 118–126. 1960567 10.1002/jemt.1060190112

[34] McLellan M. R., and Day J. G. 1995 Cryopreservation and freeze-drying protocols. Introduction. Methods Mol. Biol. 38: 1–5. 7647848 10.1385/0-89603-296-5:1

[35] Deckelbaum R. J., Shipley G. G., Small D. M., Lees R. S., and George P. K. 1975 Thermal transitions in human plasma low density lipoproteins. Science. 190: 392–394. 170681 10.1126/science.170681

[36] Robards A. W., and Sleytr U. B. 1985 Low temperature methods in biological electron microscopy. *In* Practical Methods in Electron Microscopy. Vol. 10. A. M. Glauert, editor. Elsevier, Amsterdam. 5–133.

[37] Cheng D., Mitchell D. R. G., Shieh D-B., and Braet F. 2012 Practical considerations in the successful preparation of specimens for thin-film cryo-transmission electron microscopy. *In* Current Microscopy Contributions to Advances in Science and Technology. A. Mendez-Vilas, editor. FORMATEX, Badajoz, Spain. 880–890.

[38] Tong H., Zhang L., Kaspar A., Rames M. J., Huang L., Woodnutt G., and Ren G. 2013 Peptide-conjugation induced conformational changes in human IgG1 observed by optimized negative-staining and individual-particle electron tomography. Sci. Rep. 3: 1089. 23346347 10.1038/srep01089 PMC3549606

[39] Liu Y., and Atkinson D. 2011 Immuno-electron cryo-microscopy imaging reveals a looped topology of apoB at the surface of human LDL. J. Lipid Res. 52: 1111–1116. 21460103 10.1194/jlr.M013946 PMC3090232

[40] Chatterton J. E., Phillips M. L., Curtiss L. K., Milne R. W., Marcel Y. L., and Schumaker V. N. 1991 Mapping apolipoprotein B on the low density lipoprotein surface by immunoelectron microscopy. J. Biol. Chem. 266: 5955–5962. 2005131

[41] Asano S., Fukuda Y., Beck F., Aufderheide A., Forster F., Danev R., and Baumeister W. 2015 Proteasomes. A molecular census of 26S proteasomes in intact neurons. Science. 347: 439–442. 25613890 10.1126/science.1261197

[42] von Appen A., Kosinski J., Sparks L., Ori A., DiGuilio A. L., Vollmer B., Mackmull M. T., Banterle N., Parca L., Kastritis P., 2015 In situ structural analysis of the human nuclear pore complex. Nature. 526: 140–143. 26416747 10.1038/nature15381 PMC4886846

[43] Zhang X., Zhang L., Tong H., Peng B., Rames M. J., Zhang S., and Ren G. 2015 3D structural fluctuation of IgG1 antibody revealed by individual particle electron tomography. Sci. Rep. 5: 9803. 25940394 10.1038/srep09803 PMC4419541

[44] Zhang L., Lei D., Smith J. M., Zhang M., Tong H., Zhang X., Lu Z., Liu J., Alivisatos A. P., and Ren G. 2016 Three-dimensional structural dynamics and fluctuations of DNA-nanogold conjugates by individual-particle electron tomography. Nat. Commun. 7: 11083. 27025159 10.1038/ncomms11083 PMC4820932

[45] Sandin S., Ofverstedt L. G., Wikstrom A. C., Wrange O., and Skoglund U. 2004 Structure and flexibility of individual immunoglobulin G molecules in solution. Structure. 12: 409–415. 15016357 10.1016/j.str.2004.02.011

[46] Bongini L., Fanelli D., Piazza F., De Los Rios P., Sandin S., and Skoglund U. 2004 Freezing immunoglobulins to see them move. Proc. Natl. Acad. Sci. USA. 101: 6466–6471. 15082830 10.1073/pnas.0400119101 PMC404068

[47] Lammerts van Bueren J. J., Bleeker W. K., Brannstrom A., von Euler A., Jansson M., Peipp M., Schneider-Merck T., Valerius T., van de Winkel J. G., and Parren P. W. 2008 The antibody zalutumumab inhibits epidermal growth factor receptor signaling by limiting intra- and intermolecular flexibility. Proc. Natl. Acad. Sci. USA. 105: 6109–6114. 18427122 10.1073/pnas.0709477105 PMC2329681

[48] Lammerts van Bueren J. J., Bleeker W. K., Brannstrom A., Jansson M., Peipp M., Schneider-Merck T., Valerius T., van de Winkel J. G., and Parren P. W. 2012 Retraction for Lammerts van Bueren et al., The antibody zalutumumab inhibits epidermal growth factor receptor signaling by limiting intra- and intermolecular flexibility. *Proc. Natl. Acad. Sci. USA.* **109:** 5548. 10.1073/pnas.1203736109 PMC3325735 22431606

[49] Wu G. H., Khant H. A., Chiu W., and Lee K. Y. C. 2009 Effects of bilayer phases on phospholipid-poloxamer interactions. Soft Matter. 5: 1496–1503.

[50] Hevonoja T., Pentikainen M. O., Hyvonen M. T., Kovanen P. T., and Ala-Korpela M. 2000 Structure of low density lipoprotein (LDL) particles: basis for understanding molecular changes in modified LDL. Biochim. Biophys. Acta. 1488: 189–210. 11082530 10.1016/s1388-1981(00)00123-2

[51] Cilpa-Karhu G., Jauhiainen M., and Riekkola M. L. 2015 Atomistic MD simulation reveals the mechanism by which CETP penetrates into HDL enabling lipid transfer from HDL to CETP. J. Lipid Res. 56: 98–108. 25424006 10.1194/jlr.M054288 PMC4274075

[52] Ren G., Rudenko G., Ludtke S. J., Deisenhofer J., Chiu W., and Pownall H. J. 2010 Model of human low-density lipoprotein and bound receptor based on cryoEM. Proc. Natl. Acad. Sci. USA. 107: 1059–1064. 20080547 10.1073/pnas.0908004107 PMC2798884

[53] van Antwerpen R., Chen G. C., Pullinger C. R., Kane J. P., LaBelle M., Krauss R. M., Luna-Chavez C., Forte T. M., and Gilkey J. C. 1997 Cryo-electron microscopy of low density lipoprotein and reconstituted discoidal high density lipoprotein: imaging of the apolipoprotein moiety. J. Lipid Res. 38: 659–669. 9144081

[54] Kumar V., Butcher S. J., Oorni K., Engelhardt P., Heikkonen J., Kaski K., Ala-Korpela M., and Kovanen P. T. 2011 Three-dimensional cryoEM reconstruction of native LDL particles to 16A resolution at physiological body temperature. PLoS One. 6: e18841. 21573056 10.1371/journal.pone.0018841 PMC3090388

[55] Orlova E. V., Sherman M. B., Chiu W., Mowri H., Smith L. C., and Gotto A. M. Jr 1999 Three-dimensional structure of low density lipoproteins by electron cryomicroscopy. Proc. Natl. Acad. Sci. USA. 96: 8420–8425. 10411890 10.1073/pnas.96.15.8420 PMC17531

