# Supplementary material for: Polyhedral 3D structure of human plasma very low density lipoproteins by individual particle cryo-electron tomography1
Source: J Lipid Res. 2016 Oct;57(10):1879–88. doi: 10.1194/jlr.M070375 (PMC5036368; doi:10.1194/jlr.M070375)
Supplement: Supplemental Data [file supp_57_10_1879__index.html]

Polyhedral 3D Structure of Human Plasma Very-Low-Density Lipoproteins by Individual Particle Cryo-Electron Tomography — Polyhedral 3D structure of human plasma very low density lipoproteins by individual particle cryo-electron tomography1 — Supplemental Data 

# Polyhedral 3D structure of human plasma very low density lipoproteins by individual particle cryo-electron tomography1

## Supplemental Data

- Supplemental Video S1 (.mov, 46.8 MB) - Supporting Video S1. Movie of 3D reconstruction procedures of human plasma VLDLs by IPET. The movie includes the tilt series of survey views of VLDL particles by cryo-electron tomography; IPET 3D reconstruction of a representative VLDL particle and a VLDL-mAB particle. The tilt series were collected from -63o to +63o in 1.5o steps.
